# Strain-level diversity of commercial probiotic isolates of *Bacillus*, *Lactobacillus*, and *Saccharomyces* species illustrated by molecular identification and phenotypic profiling

**DOI:** 10.1371/journal.pone.0213841

**Published:** 2019-03-22

**Authors:** Juliana M. Ansari, Christine Colasacco, Elli Emmanouil, Scott Kohlhepp, Olivia Harriott

**Affiliations:** Department of Biology, Fairfield University, Fairfield, Connecticut, United States of America; Universita degli Studi di Parma, ITALY

## Abstract

Probiotic products are becoming more prevalent as awareness of the role of beneficial microbes in health increases. Ingredient labels of these products often omit identifications at the strain level, making it difficult to track down applicable published research. In this study, we investigated whether products labeled with the same species name contained different strains of those species. From 21 commercially available probiotic supplements and beverages, we cultured five main species: *Bacillus coagulans*, *Bacillus subtilis*, *Lactobacillus plantarum*, *Lactobacillus rhamnosus*, and the yeast *Saccharomyces boulardii*. To confirm the identity of each bacterial isolate, we applied standard molecular approaches: 16S rRNA gene sequencing and Matrix Assisted Laser Desorption Ionization Time-of-Flight mass spectrometry (MALDI-TOF MS). Phenotypic profiling and identification were performed with the Biolog Microbial Identification system. All of the bacterial isolates were correctly identified by at least one approach. Sequencing the 16S rRNA gene led to 82% of species identifications matching the product label, with 71% of isolates identified by MALDI-TOF MS and 60% identified correctly with the Biolog system. Analysis of the Biolog phenotypic profiles revealed different patterns of carbon source usage by each species, with sugars preferentially utilized by all except *B*. *subtilis*. To assess the strain-level differences, we compared strains of the same species and found variability in carbohydrate utilization and tolerance to environmental stressors (salt, acidity, antibiotics). By demonstrating that products listing the same species often contain strains with different 16S sequences and phenotypes, this study highlights that current labels of probiotic supplements do not sufficiently convey the strain diversity in these products.

## Introduction

A widespread awakening in the public and medical community’s interest in beneficial bacteria for promoting health is underway. Accelerated by the ease and affordability of rapid DNA sequencing technology, an avalanche of studies in animal models and humans has linked the microbiome (the microbial community inhabiting the human body) body to a wide range of diseases. This mounting knowledge of the human microbiome has stimulated interest in bacteria that confer a health benefit to the host (probiotics) or foods that selectively enhance growth of certain beneficial microbes (prebiotics). While fermented foods and beverages are common in the traditional diets around the world, market demand for probiotic foods is growing [1][2]. Probiotic strains are typically selected from lacto-fermented foods or the human digestive tract, then studied clinically for health-promoting effects. Two predominant groups of probiotic bacteria are the lactic acid bacteria (*Lactobacillus* and related genera) and bifidobacteria (*Bifidobacterium* spp). Certain *Bacillus* species and the yeast *Saccharomyces boulardii* are also sold in probiotic supplements and beverages. Candidate probiotic bacteria such as *Akkermansia municiphila* and *Faecalibacterium prausnitzii*, often termed “next generation probiotics,” have been identified from human microbiome studies but are not yet commercially available [3].

The promoted advantage of probiotics is the maintenance or restoration of the balance between pathogens and healthy necessary bacteria, via mechanisms such as reducing permeability of the intestinal epithelium, binding to pathogens, competing for nutrients, antimicrobial production, and modulating the immune system [4][5]. Further benefits include the production of enzymes and bioactive compounds. Lactic acid bacteria produce metabolites including B vitamins, gamma-aminobutyric acid (GABA), bioactive peptides, bacteriocins, and other complex molecules such as exopolysaccharides [6]. Among beneficial effects on digestive health [7], probiotics have been shown prevent or reduce symptoms of traveler’s diarrhea [8] and antibiotic-associated diarrhea [9]. Symptoms of irritable bowel syndrome (IBS) may also be alleviated by specific probiotic bacteria [10][11]. For other bowel diseases, authors of a recent systematic review concluded that the evidence for probiotics was stronger for ulcerative colitis than for Crohn’s disease [12]. Another evidence-based review analyzing clinical trials through June 2018 concluded that the most thoroughly supported benefit of probiotics was for treatment of pediatric acute diarrhea with the highest number of randomized controlled trials [13].

Despite a growing number of clinical trials supporting the specific benefits of well-established strains [11][14], more carefully designed and controlled studies are needed [15][16]. There is a gap between the advertised benefits of probiotics and the evidence to support their efficacy, due in part to the limited regulation on probiotic supplements. In the United States, probiotics are currently categorized by the Food and Drug Administration (FDA) as food additives or ingredients. The microbial strains they contain are classified as “GRAS” (generally recognized as safe), but are not under stringent regulatory scrutiny and do not need to show proof of efficacy. Though many probiotic strains are evaluated for specific beneficial effects in clinical trials, the strain-level identification (ID) is not always provided on the ingredient labels of supplements [17]. In some cases probiotic properties are species- or genus-wide [18], however omitting the strain information raises several concerns, including quality control and safety [17][19], as well as misinformation and the possibility that the strain does not actually possess the probiotic effects of clinically verified strains [20]. Probiotic properties can also change under different manufacturing conditions. A study comparing 15 isolates of *L*. *rhamnosus* strain GG from different manufacturers found that the isolates varied in their ability to compete with pathogens via adhesion to human intestinal mucus [21].

The objective of the present study, carried out partially within an undergraduate microbiology lab course, was to investigate whether products that listed the same species name on the label, actually contained different strains of those species. We looked for strain-specific differences by characterizing the physiology and metabolism of these bacteria (and yeasts) using phenotypic profiling. Clarifying the physiology of beneficial microbes can enhance the potential therapeutic value of probiotic products [22][23], since the end-products of microbial metabolism, such as lactic acid or short-chain fatty acids (SCFA) often contribute to their health-promoting effect. Many experimental methods can be used to characterize microbial metabolic pathways that may tie into expression of probiotic phenotypes. Proteomics approaches [24], transcriptomics and metabolic flux analyses are useful strategies for harnessing and/or engineering metabolic pathways to improve probiotic strain performance [25]. To ensure an accessible supply of appropriate carbon sources to lead to desired metabolites, specific foods may be consumed directly in the diet or prebiotic fibers can be added via supplements. An understanding of the nutritional preferences of beneficial bacteria will improve translation into effective products, such as “synbiotics” that contain both probiotics and prebiotics [26][7].

In the current study, we isolated pure cultures from 21 commercial probiotics, confirmed bacterial identifications with standard molecular methods, then investigated the phenotypic differences between microbes from each product with the Biolog Microbial Identification system, a plate-based assay consisting of 71 carbon source utilization tests and 23 chemical sensitivity tests [27]. We hypothesized that comparing the phenotypic profiles of these common probiotic microbes side-by-side would reveal differences in carbon source utilization and chemical sensitivity–not only between different probiotic species, but potentially among strains of the same species that were isolated from different brands.

## Materials and methods

### Isolation of bacteria from commercial probiotic products

Probiotic products were purchased in 2016 and 2017 from the online retailer Amazon.com, or drugstores or grocery stores in Shelton, Connecticut, USA and Fairfield, Connecticut, USA (geographical coordinates 41.14° N, 73.26° W). A list of products is provided in S1 Table. Bottles were opened within one month of the date of purchase and stored at 4#x00B0;C. “Single-strain” probiotics contained only one species of live microorganism, and “Multi-strain” probiotics contained two or more species listed on the label. Contents of each probiotic capsule were aseptically emptied into a microcentrifuge tube containing 1 ml of sterile water and mixed thoroughly. Tablets were ground with a sterilized mortar and pestle and combined with sterile water. Probiotic beverages were sampled directly from the original bottle. Using a sterile swab or inoculating loop, each sample was streaked for isolation onto the surface of the appropriate agar growth medium. The following culture media were used: MRS (de Man, Rogosa, and Sharpe) agar for *Lactobacillus* species and *Bacillus coagulans*, TSA (Tryptic Soy Agar) for *Bacillus subtilis*, and SDA (Sabouraud Dextrose Agar) for yeast isolates. Agar plates were incubated at 30–33#x00B0;C for 48–72 hours aerobically, and individual isolated colonies were selected, and re-streaked for isolation prior to storing as a frozen glycerol stock. Bacteria were Gram stained following the standard procedure [28] and viewed under oil immersion with the 100x objective lens. As a preliminary differentiation step between *Lactobacillus* and *Bacillus*, endospore staining was performed on 1-week old cultures following the Schaeffer-Fulton procedure [28].

### PCR amplification and sequencing of 16S rRNA gene

Polymerase Chain Reaction (PCR) was performed using DNA obtained directly from bacterial colonies. To amplify the near full-length 16S rRNA gene, the primers 27F (5’-AGAGTTTGATCCTGGCTCAG-3’) and 1492R (5’-GGTTACCTTGTTACGACTT-3’) were used. On ice, a Master mix was prepared, containing water, buffer, MgSO4, dNTPs, and primers following the manufacturer’s instructions for HotStart KOD Polymerase (Millipore Sigma, St. Louis, MO). Aliquots of 50 μl of the Master mix were added to labeled 8-strip PCR tubes, and a sterile 100 μl pipette tip was used to pick up a pinpoint amount of a bacterial colony and transfer the cells directly into the appropriate PCR tube. Amplification reactions were run on a Bio-rad Thermocycler (Bio-rad, Hercules, CA) using the following cycling conditions: denaturation at 95#x00B0;C for 2 minutes, 35 cycles of: [95#x00B0;C denaturation for 20 seconds, 48#x00B0;C annealing for 20 seconds, 70#x00B0;C extension for 35 seconds], followed by a final extension step at 70#x00B0;C for 3 minutes. After visualizing the 1450-bp amplicons from each PCR reaction using gel electrophoresis, the PCR amplicons were purified using the QIAquick PCR Purification Kit (Qiagen, Valencia, CA). The DNA purified from the PCR reactions was quantified using a Take3 micro-volume plate with the Gen5 Microplate Reader (Biotek Instruments, Winooski, VT). DNA samples were prepared with forward or reverse primer and PCR products were sequenced by Sanger sequencing at the Yale University DNA Analysis Facility on Science Hill, using an Applied Biosystems Genetic Analyzer (New Haven, CT). The .abi files were downloaded and DNA chromatograms were viewed and trimmed using Geneious bioinformatics software (http://www.geneious.com/). Resulting forward and reverse sequences were searched against sequences in the Genbank non-redundant (nr) nucleotide database using Standard Nucleotide BLAST (blastn), and the top-scoring hits were recorded for each organism (see S2 Table).

### Matrix-assisted laser desorption ionization time-of-flight (MALDI-TOF) mass spectrometry

Bacteria from frozen stock cultures were transferred to MRS or TSA plates and incubated at 30°C for 48 hours prior to identification. Proteins were extracted using either the on-target method or by using an ethanol-formic acid protocol described by Friewald and Sauer [29]. Cells from isolated colonies were directly smeared onto a disposable FlexiMass DS target plate using a sterile toothpick. One μl of 25% formic acid was added to the spot and allowed to air dry followed by the addition of 1 μl of the α-Cyano-4-hydroxycinnamic acid (CHCA) matrix solution. The CHCA matrix solution contained 50 mg of CHCA dissolved in a 33/33/33 mixture of acetonitrile/ethanol/dH2O containing a final concentration of 3% trifluoroacetic acid. When the on-target method yielded spectra with poor resolution, proteins were extracted prior to spotting using ethanol and formic acid [29]. Cells from colonies were dissolved in 300 μl of dH_2_O and inactivated by adding 900 μl of room temperature absolute ethanol. The cell suspension was centrifuged twice at 10,000 x g for 2 minutes to remove the supernatant. The pellet was air dried at room temperature for 1 minute and dissolved in 10 μl 70% formic acid. Ten μl acetonitrile was added to the formic acid-cells mixture followed by centrifugation at 10,000 x g for 2 minutes at room temperature. The resulting supernatant containing extracted proteins was transferred to a separate tube. One μl of the supernatant was spotted onto the target plate and overlaid with 1 μl of the matrix.

MALDI-TOF MS was performed on the AXIMA Confidence iDplus MALDI-TOF Mass Spectrometer (Shimadzu) using Launchpad software version 2.9.1 and the VITEK MS Plus Spectral Archive and Microbial Identification System (SARAMIS) database, V4.12. Samples were analyzed in the positive linear mode with a laser frequency of 50 Hz and within a mass range of 2000–20,000 Da. The acceleration voltage was 20 kV and extraction delay time 200 ns. Spectra were generated from 500 laser shots and each target plate was calibrated before samples were analyzed using *Escherichia coli* DH5alpha. Samples were run at least in duplicate and spectra acquired by Launchpad were processed by SARAMIS. Each spectrum was assigned a confidence level based on a comparison to SuperSpectra in the SARAMIS database. SARAMIS does not assign a taxonomic name if the confidence levels are below 75%.

### Biolog identification and metabolic profiling

Freshly grown (24–48 hr) colonies, from TSA or MRS agar, were used for bacterial identification on GenIII microplates with the Biolog semi-automated system (Biolog Inc., Hayward, CA) following the manufacturer’s instructions. The quality control Gram-positive strains (*Paenibacillus polymyxa* ATCC 842 and *Staphylococcus epidermidis* ATCC 12228) were used to test the performance of the Biolog GenIII Microplates prior to testing probiotic isolates. Bacteria were added to the recommended inoculating fluid and transmittance (T) was measured and adjusted to 90–98% T. For all *Lactobacillus* species, Inoculating Fluid C (IF-C) was used. For *Bacillus* species, either Inoculating Fluid A or B (IF-A or IF-B) was used. Details of the experimental conditions can be found in S3 Table. Cells suspended in IF were dispensed with an automatic multichannel pipettor into the GenIII 96-well microplate (100 μl per well). The GenIII microplates were incubated at 33#x00B0;C for 16–48 hours and read using the MicroLog plate reader and associated software (Biolog Inc., Hayward, CA) once the positive control well A10 turned purple (typically at 20–24 hr of incubation). Positive growth responses are indicated by a color change based on redox dye chemistry. Identification is made by the GenIII MicroStationsoftware, which compares the phenotypic fingerprint with a fingerprint database of known bacteria [30]. Similarity (SIM) scores are assigned reflecting how well the isolate matches the pattern in the database, and an identification is given if the SIM score is >0.5. Plate images of each isolate were saved for later analysis, along with the GenIII Microplate reference pattern for each species. Results from all Biolog plates were transcribed into a single summary table, using a “P” for positive reaction wells (purple on MicroLog software), representing growth and utilization of a carbon source. Wells scored by the software as borderline (half-moon on MicroLog software, could be positive or negative) [30] were recorded as “h” for half. A total of 24 wells (Row G1-G9, H1-H9, and column 9) were omitted from the final results charts because few strains had positive growth in those wells.

For the Biolog assay of yeast, the quality control yeast *Candida albicans* ATCC 10231 was used to test the performance of the YT MicroPlates. Yeast strains isolated from probiotic products were grown for 48–72 hour on SDA at 26#x00B0;C, then inoculated into 10 ml sterile water as specified in the instructions for use. The cell suspension was adjusted to 50% transmittance, then pipetted into the wells of a YT microplate. YT plates were incubated at 26#x00B0;C for 24–72 hours, and analyzed at 24, 48, and 72 hours using the MicroLog plate reader (using the yeast YT database) until an identification was made.

### Antibiotic susceptibility testing

The Kirby-Bauer test for antibiotic susceptibility was followed with minor modifications. Mueller Hinton agar was used for *Bacillus subtilis* and MRS agar used for *Lactobacillus* spp. Using sterile swabs, overnight liquid cultures were spread in a zig-zag pattern to create a “lawn” of growth on large 150-mm agar plates. A 12-place BD BBL Sensi-Disc Dispenser was used to deposit the following antibiotic susceptibility Sensi-discs (BD, Franklin Lakes, NJ) onto the agar surface: Ampicillin (AM10), Bacitracin (B10), Chloramphenicol (C30), Ciprofloxacin (CIP5), Erythromycin (E15), Gentamicin (GM10), Kanamycin (K30), Neomycin (N30), Penicillin (P10), Streptomycin (S10), Tetracycline (Te30), and Vancomycin (VA30). After 24 hours of incubation at 33#x00B0;C, zones of inhibition were measured and diameters compared to the reference Zone Diameter Interpretive Chart, updated by the National Committee for Clinical Laboratory Standards, accessed in [28]. Susceptibility to each antibiotic was recorded as susceptible (S), resistant (R), or intermediate (I) based on the diameter of the zone of inhibition in the reference chart.

### Probiotic product label analysis

To estimate the percentage of products listing specific strains on the label, products were evaluated from four marketplaces: a major online retailer, two drugstore chains, and a retail superstore with brick-and-mortar store locations in Shelton, Connecticut, USA. A search for products from the online retailer was conducted between January-April 2017 using the keyword “probiotic” and the names of the following probiotic microbes: *Bacillus coagulans*, *Bacillus subtilis*, *Lactobacillus acidophilus*, *Lactobacillus gasseri*, *Lactobacillus plantarum*, *Lactobacillus rhamnosus*, *Bifidobacterium*, and *Saccharomyces boulardii*. At least 20 products for each organism were checked, and if the label image contained a specific strain name or number, this was recorded. For brick-and-mortar stores (visited in April 2018), we counted unique products on the shelves in the probiotic section, examined the labels, and recorded the number of products that listed at least one strain ID on the label. In stores with a high volume of store-brand generic products, those products were only counted individually if the listed organisms were distinct from another store-brand product. Note that products found on the shelves at multiple stores were tallied each time in the count for that store, so the list for each store includes overlapping products (particularly the most popular name-brand probiotics).

## Results and discussion

### Isolation of microbes from probiotics

**Fig 1:**
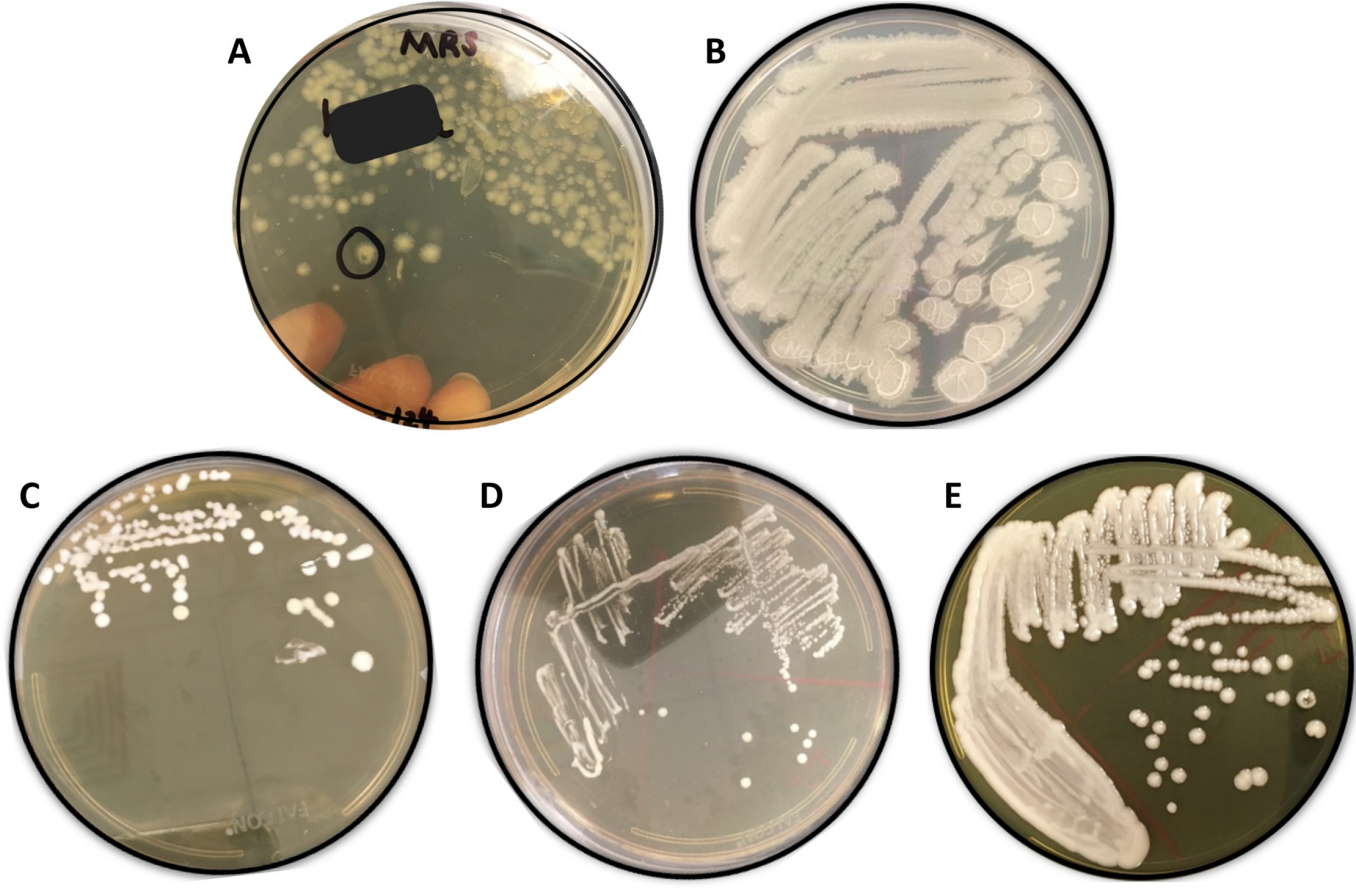
Typical colony morphologies of probiotic isolates grown on agar media. (A) *Bacillus coagulans* on MRS agar, (B) *Bacillus subtilis* on TSA, (C) *Lactobacillus plantarum* on MRS agar, (D) *Lactobacillus rhamnosus* on MRS agar, (E) *Saccharomyces boulardii* on SDA agar.

We selected a variety of probiotic supplements and beverages to adequately represent the selection of products available to consumers. We isolated microbes from 21 commercially available probiotic products, of which only seven products listed the strain-level identification. Pure cultures of bacteria and yeast were isolated from 15 probiotic supplements, six probiotic beverages, and four environmental sources (Chaas fermented beverage, fruit fly gut, kale, and leaves) to serve as “wild” microbes for comparison. Though the lactobacilli are fermentative organisms, the aerobic growth of these species was nearly the same as growth with CO_2_ Gaspaks, so further experiments were conducted under aerobic conditions. Attempts to culture the obligately anaerobic *Bifidobacterium* species using anaerobic jars were not successful, so *Bifidobacterium* spp. were excluded from this investigation due to the lack of an anaerobic culturing system in the laboratory facilities available. The main species cultured were the bacteria *Bacillus coagulans*, *Bacillus subtilis*, *Lactobacillus plantarum*, and *Lactobacillus rhamnosus*, and the yeast *Saccharomyces boulardii*. Typical colony morphologies of these isolated microbes on MRS agar are shown in Fig 1.

While the *Lactobacillus* colonies had very similar appearances (off-white to white, circular, creamy), the colony morphology of each *Bacillus* species was distinct. *B*. *coagulans* colonies had irregular edges, a translucent tan color, and slower growth. The colonies of *B*. *subtilis* displayed rapid, spreading growth, were opaque off-white in color, raised, and wrinkled. Gram staining confirmed that all bacteria were Gram-positive rods. Yeast cell morphology was confirmed by crystal violet staining. As expected, endospore staining revealed that only the *Bacillus* species formed spores (not shown). *B*. *coagulans* formed visibly stained spores only when the bacteria were grown on TSA, not on MRS. In one single-strain probiotic (strain code 8) labeled as “*Bacillus coagulans*,” both *B*. *coagulans* and *B*. *subtilis* were cultured repeatedly from the original supplement, suggesting possible contamination within the original product.

### Molecular identification

After isolating pure cultures from commercially available probiotics, we used three approaches to identify the bacteria from these products. Table 1 summarizes the number of identifications obtained using each method. Identifications were considered accurate if they matched the species name listed on the ingredient label. All of the bacterial isolates were identified correctly by at least one of the three methods. The standard molecular method of 16S gene sequencing performed better, providing correct identifications for a higher percentage of bacteria than MALDI-TOF mass spectometry or the Biolog assay (Table 1), however a Chi-squared test indicated that there was not a statistically significant difference between the observed and expected total % correctly identified by each method (X^2^ = 2.203, p = 0.332).

**Table 1:** Number of species identifications matching probiotic ingredient label.

	*Bacillus coagulans* (n = 4)	*Bacillus subtilis* (n = 3)	*Lactobacillus plantarum* (n = 4)	*Lactobacillus rhamnosus* (n = 4)	Other *Lactobacillus* spp. (n = 2)	*Saccharo-myces boulardii* ^a^ (n = 3)	Total^b^
16S sequencing	3 (75%)	2 (67%)	4 (100%)	3 (75%)	2 (100%)	n/a	14/17 (82%)
MALDI-TOF	2 (50%)	3 (100%)	3 (75%)	4 (100%)	0 (0%)	n/a	12/17 (71%)
Biolog	0 (0%)	3 (100%)	4 (100%)	3 (75%)	0 (0%)	2 (67%)	12/20 (60%)
Correct ID^c^	4 (100%)	3 (100%)	4 (100%)	4 (100%)	2 (100%)	2 (67%)	

Number and (percent) of isolated strains that were correctly identified as the species listed on the probiotic label. Each method was performed in duplicate. The difference between the observed and expected total % identified by each method was not statistically significant (Chi-Squared test, X^2^ = 2.203, p = 0.332).

^a^Yeast identification was only performed with Biolog method.

^b^Bacteria were isolated from 17 probiotic products, and yeast isolated from 3 products (for which ingredient label listed *S*. *boulardii*), bringing the Biolog total to 20.

^c^Isolated strains identified correctly by at least one of the three identification approaches.

The reliability of the Biolog method varied between species, yielding correct identifications for all of the *B*. *subtilis* and *L*. *plantarum* isolates but none of the *B*. *coagulans* strains. Because these identification techniques were performed in some cases by operators (students) with minimal laboratory experience, the accuracy may be lower than would be expected for experienced technicians. However, the number of non-matching identifications can also be attributed to limiting factors such as the extent of species coverage in each database (lowest for Biolog), poor growth of certain strains, and potentially overlapping classifications between closely related species, particularly within the genus *Lactobacillus* [31].

The taxonomic identifications obtained with each method, for each isolate, are listed in Table 2. Results for yeast isolates (identified with Biolog only) are also shown. Each isolate was assigned a code number to de-identify the product brand, with products classified as either “single-strain” or “multi-strain” based on the microbes listed on the ingredient label. Seven of the products listed the strain-level identification (Strain ID) on the label, with three products containing *B*. *coagulans* GBI-30 6086, and one each listing *B*. *subtilis* DE111, *L*. *plantarum* 299V, *L*. *rhamnosus* GG, and *L*. *rhamnosus* LCR35 (Table 2). Table 2 includes a total of 26 isolated organisms: the 20 microbes for which the species was listed on a label, plus six microbes for which the identity was unknown prior to analysis. The latter were designated ENV for “environmental” and were isolated either from probiotic beverages (kombucha, Chaas fermented dairy beverage) or environmental sources (*Drosophila* fruit fly gut, surfaces of leaves).

**Table 2:** Species identifications of probiotic microbes using three methods.

Source	Code	Probiotic Label	16S sequence BLAST Result	MALDI-TOF Result	Biolog Result
***Bacillus coagulans***					
Probiotic Beverage	5	*Bacillus coagulans GBI-30 6086*	*Bacillus coagulans*	*Bacillus coagulans*	No ID
Probiotic Beverage	6	*Bacillus coagulans GBI-30 6086*	*Bacillus coagulans*	*Bacillus subtilis*	*Sporolactobacillus kofuensis*
Single-strain Probiotic	8	*Bacillus coagulans*	*Bacillus subtilis*^a^	*Bacillus coagulans*	*Bacillus subtilis*^a^*; L*. *paracasei*
Single-strain Probiotic	14	*Bacillus coagulans GBI-30 6086*	*Bacillus coagulans*	*Lactobacillus pentosus/plantarum*	*Brochothrix campestris*
***Bacillus subtilis***					
Single-strain Probiotic	9	*Bacillus subtilis*	*Bacillus* sp. strain BCBT29	*Bacillus subtilis*	*Bacillus atrophaeus/subtilis*
Multi-strain Probiotic	13	*Bacillus subtilis*	*Bacillus subtilis*	*Bacillus subtilis*	*Bacillus subtilis*
Multi-strain Probiotic	15	*Bacillus subtilis* DE111	*Bacillus subtilis*	*Bacillus subtilis*	*Bacillus subtilis*
ENV: leaf surface	20	N/A (environmental)	N/A	N/A	*Bacillus atrophaeus/subtilis*
***Lactobacillus plantarum***					
Single-strain Probiotic	2	*Lactobacillus pentous/plantarum*	*Lactobacillus plantarum*	*Lactobacillus pentosus/plantarum*	*Lactobacillus plantarum*
Multi-strain Probiotic	11	*Lactobacillus plantarum complex*	*Lactobacillus plantarum*	No ID	*Lactobacillus plantarum*
Multi-strain Probiotic	12	*Lactobacillus plantarum*	*Lactobacillus plantarum*	*Lactobacillus plantarum*	*Lactobacillus plantarum*
Probiotic Beverage	16	*Lactobacillus plantarum 299V*	*Lactobacillus plantarum*	*Lactobacillus pentosus/plantarum*	*Lactobacillus plantarum*
ENV: Fruit fly gut	19	N/A (environmental)	*Lactobacillus plantarum*	N/A	*Lactobacillus plantarum*
***Lactobacillus rhamnosus***					
Single-strain Probiotic	1	*Lactobacillus rhamnosus* GG	*Lactobacillus rhamnosus* GG	*Lactobacillus rhamnosus*	*Weissella viridescens*
Multi-strain Probiotic	7	*Lactobacillus rhamnosus*	*Staphylococcus epidermidis*^*b*^	*Lactobacillus rhamnosus*	*Lactobacillus rhamnosus*
Single-strain Probiotic	10	*Lactobacillus rhamnosus* LCR35	*Lactobacillus rhamnosus*	*Lactobacillus rhamnosus*	*Lactobacillus rhamnosus*
Multi-strain Probiotic	17	*Lactobacillus rhamnosus*	*Lactobacillus rhamnosus*	*Lactobacillus rhamnosus*	*Lactobacillus rhamnosus*
ENV: fermented milk beverage "Chaas"	18	N/A (environmental)	*Lactobacillus casei*	*Lactobacillus sp*.	*Lactobacillus rhamnosus*
**Other *Lactobacillus spp*.**					
Single-strain Probiotic	3	*Lactobacillus gasseri*	*Lactobacillus gasseri*	No ID	S*treptococcus oralis*
Single-strain Probiotic	4	*Lactobacillus acidophilus*	*Lactobacillus acidophilus*	No ID	No ID
**Yeasts**					
Kombucha 1	21	*Saccharomyces boulardii*	N/A	N/A	*Saccharomyces cerevisiae a/Tor*.*pretorein*
Kombucha 2	22	N/A	N/A	N/A	*Pichia chmeri A*
Kombucha 3	23	N/A	N/A	N/A	*Hanseniospora guillermondii/uvarum*
Probiotic rice wine	24	*Saccharomyces boulardii*	N/A	N/A	*Saccharomyces boulardii*
Multi-strain probiotic	25	*Saccharomyces boulardii*	N/A	N/A	*Saccharomyces boulardii*
ENV: Kale leaf	26	N/A (environmental)	N/A	N/A	*Sporidiobolus pararoseus A*

ENV: Environmental isolate; No ID: no identification after three repeats; N/A: not applicable (Species name not listed or method not performed). Top-scoring nucleotide BLAST hits for 16S sequences are shown in S2 Table.

^a^ contamination identified in the original capsule (labelled *B*. *coagulans*, contained *B*. *coagulans* and *B*. *subtilis)*

^b^ contamination was likely introduced during PCR

Species identifications obtained by the three methods were in agreement for the majority of isolates. Sequencing of the 16S rRNA gene has become an established molecular identification technique since its introduction in the 1980s [32], but the sequence is not variable enough to distinguish among strains of the same species. The universal primers 27F and 1492R were used to amplify the near full length 16S rRNA gene, using direct colony PCR. In most cases, the nucleotide BLAST search of the forward and the reverse sequences yielded the same species, but different strain IDs (S2 Table). For the seven products listing the strain IDs on the labels, the top-scoring BLAST hit of the 16S sequence usually did not match the exact strain names, with the exception of *L*. *rhamnosus* strain GG (S2 Table). None of the top-scoring nucleotide BLAST hits were identical across the bacterial strains isolated from different products (S2 Table), indicating sequence variation between each isolate. Several of the conflicting identifications (strain codes 7 and 8) are attributed to contamination during the procedure (Table 2).

Using the second molecular approach, MALDI-TOF mass spectrometry, the mass fingerprints of 15 bacteria isolated from commercial probiotics (or environmental sources) were obtained and identified. Only peptide mass spectra matching the database with confidence scores >75% were assigned an identification with the SARAMIS software. Confidence interval scores for each isolate are provided in S4 Table. For 12 of the strains, the MALDI identification matched that of the probiotic label (Table 1). The “Chaas” isolate, cultured from a homemade fermented milk beverage, was identified by MALDI-TOF MS only at the genus level (*Lactobacillus* sp.). All *B*. *subtilis* isolates were correctly identified by MALDI-TOF, but the *B*. *coagulans* strains were not as consistently identified by this technique (Table 2). Several *Lactobacillus* strains were unable to be identified: *L*. *acidophilus*, *L*. *gasseri*, and one *L*. *plantarum* (strain 11.) However, other *L*. *plantarum* isolates were successfully identified with confidence scores between 77–88% (S4 Table).

Other research groups have applied these approaches to identify probiotic *Lactobacillus* and *Bacillus* species. In one study of *Lactobacillus* species, MALDI mass spectrometry performed better in identifying species at the subspecies, or strain level than either 16S sequencing or Biolog identification [33]. However, a limitation with the MALDI approach is that the peptide mass spectra must exist in the database [34]. Another study of 148 strains of *Lactobacillus* species isolated from food, reported that the MALDI approach led to accurate species identifications more often than 16S PCR (93% accuracy vs. 77% for PCR) [35]. Sato and colleagues (2017) used MALDI-TOF and repetitive sequence based PCR (rep-PCR) for rapid strain typing of strains of *B*. *coagulans* [36]. This group found a strong correlation between these two methods to successfully distinguish between closely related strains, and reported that carbohydrate utilization patterns correlated well with the MALDI and rep-PCR results for some phylogenetic clusters [36].

Public health laboratories or clinical researchers tracking the epidemiology of infectious agents have traditionally used other molecular approaches to distinguish bacteria at the strain level, such as multi-locus sequence typing (MLST), pulsed-field gel electrophoresis (PFGE), or amplified fragment length polymorphism (AFLP) analysis [37][38]. PFGE and AFLP have been used to differentiate among probiotic strains of *L*. *rhamnosus* [39] and *L*.*plantarum* isolated from various sources [40]. Ceapa and colleagues (2015) identified genotypic clusters of *L*. *rhamnosus* with AFLP that correlated with functional metabolic clusters determined by Biolog profiling [41]. However, these molecular approaches for strain typing are being displaced by whole-genome sequencing (WGS), due to its decreasing costs, improved efficiency and accuracy [42].

### Biolog identification and phenotypic profiling

Ten of the bacterial isolates from probiotics were correctly identified using the Biolog Microbial ID system (Table 1), and two of three yeasts were correctly identified as *S*. *boulardii* (Tables 1 and 2). Among the *Lactobacillus* isolates, *Lactobacillus plantarum* strains were the most amenable to identification with the Biolog assay. All of the *L*. *plantarum* strains isolated from probiotic products were correctly identified (S3 Table). One *L*. *rhamnosus* strain yielded an incorrect identification, *Weissella viridescens*. Neither *L*. *acidophilus* nor *L*. *gasseri* were identified correctly with the Biolog assay. For the *Bacillus* strains isolated from probiotics, three *Bacillus subtilis* isolates were readily identified using the Biolog system, while none of the three *B*. *coagulans* isolates came up as *B*. *coagulans* (Table 2, S3 Table). It is possible that this is due poor growth prior to the Biolog assay or the choice of suboptimal inoculating fluid (IF-A/IF-B rather than IF-C) for correct identification in the Biolog GenIII database.

**Fig 2:**
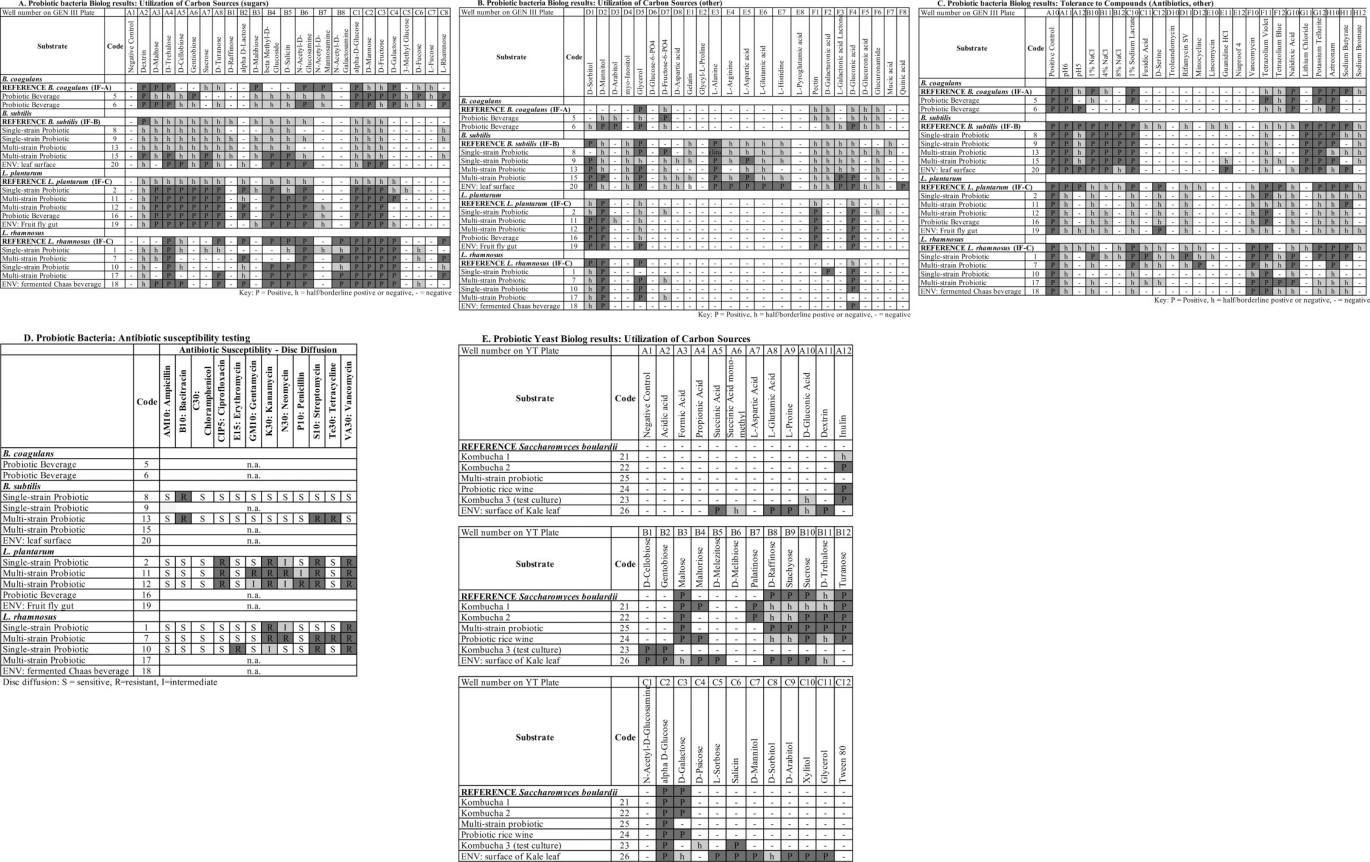
Phenotypic profiling of probiotic bacteria and yeast. (A) Probiotic bacteria Biolog results: Utilization of carbon sources (sugars). (B) Probiotic bacteria Biolog results: Utilization of carbon sources (other). (C) Probiotic bacteria Biolog results: Tolerance to compounds (Antibiotics, other). (D) Probiotic bacteria: Antibiotic susceptibility testing. Key for Disc diffusion: S = sensitive, I = intermediate, R = resistant. (E) Probiotic yeast Biolog results: Utilization of carbon sources. Wells of the yeast (YT) plate in Fig 2E contain different carbon sources than the same numbered wells of the Biolog GenIII plate for bacteria. Key for Biolog wells: P = positive, h = half (borderline positive/negative), (-) = negative. Note: only 47 of the 71 carbon sources on the GenIII plate are displayed in Fig 2A and 2B; the wells G1-8, H1-8, and Column 9 were mostly negative and are not shown.

Fig 2 depicts the aggregated results from all Biolog plates, grouped by species. Carbon source utilization patterns are shown in Fig 2A and 2B and tolerance to environmental stressors (acidity, salt, and various compounds) shown in Fig 2C. Strain-specific differences were observed for all of the species, for both carbon source utilization and chemical sensitivity tests (Fig 2 and S5 Table). The reference pattern for each species, from the Biolog GenIII database, is displayed in the top row of each section. Dark gray “P” wells indicate a strong positive, while the lighter “h” wells indicate borderline results; the preferred carbon sources are typically used up more rapidly and completely, yielding a dark purple well, while the less preferred substrates are used more slowly and incompletely [30].

**Fig 3:**
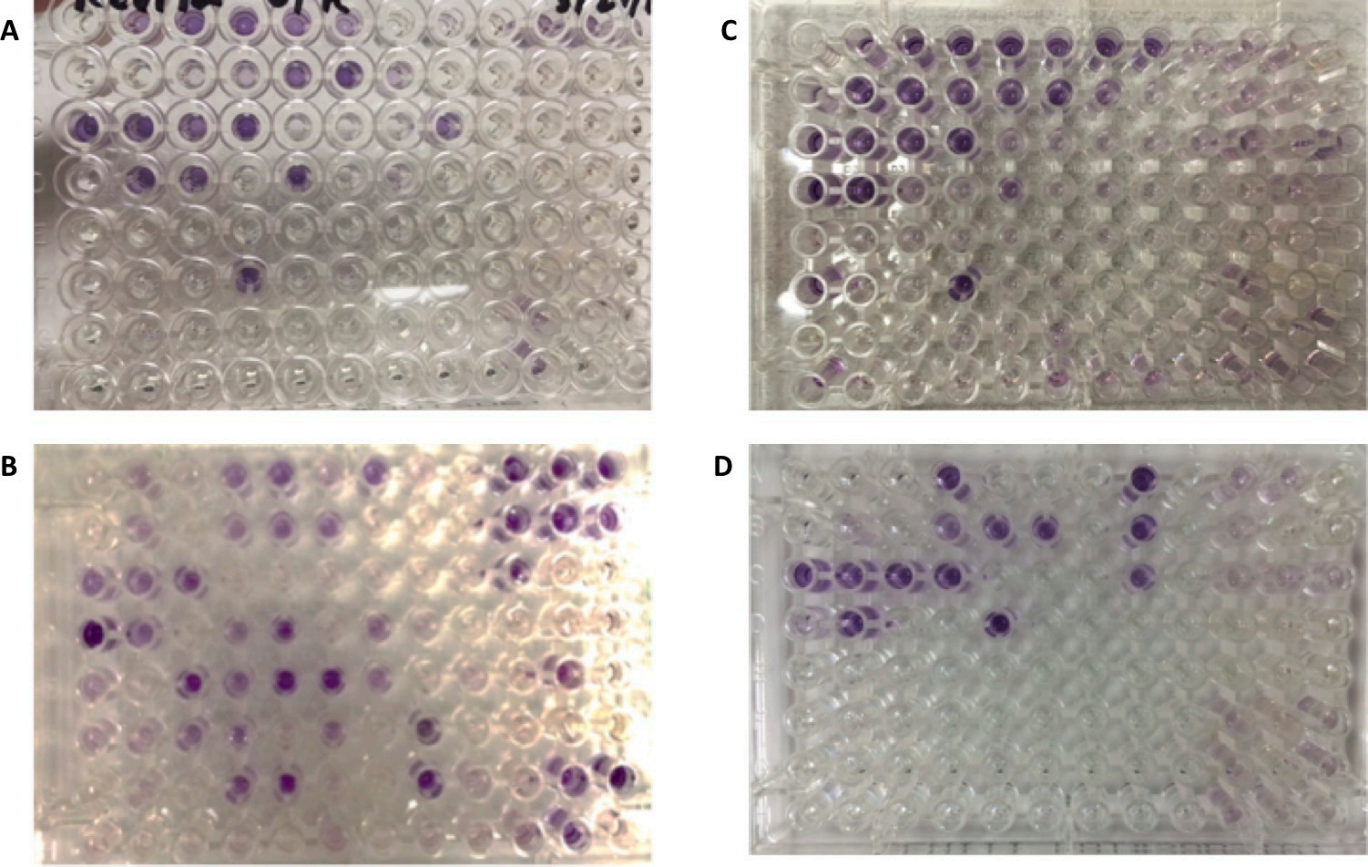
Representative biolog plates. (A) *Bacillus coagulans* (no Biolog ID), (B) *Bacillus subtilis*, (C) *Lactobacillus plantarum*, (D) *Lactobacillus rhamnosus*.

Fig 3 displays representative Biolog plates for the four bacterial species after incubation, with purple wells indicating positive growth.

Even without yielding a correct microbial identification, Biolog phenotypic profiling was a valuable strategy to uncover evidence of strain-level diversity between the isolates. Comparing the carbon source utilization patterns of the *Lactobacillus* and *Bacillus* species revealed the shared and unique properties of these probiotic bacteria (Fig 2A and 2B). A striking difference between the two genera is that the *Lactobacillus* species display a stronger preference for sugars. All of the *Lactobacillus plantarum* strains had a consistent pattern of sugar utilization, with a positive reaction in the same 14 sugars for the five strains tested. These results correlate well with comparative functional genomics and metabolic profiling studies on *L*. *plantarum* [40][43]. *L*. *plantarum* strains, but not *L*. *rhamnosus*, utilized the complex polysaccharide, pectin (Fig 2B, well F1), which is found in fruits. The environmental isolate of *L*. *plantarum* from a fruit fly gut utilized pectin as well. Pectinolytic enzymes have been characterized in *L*. *plantarum* [44] and pectin affects the probiotic phenotype of this species *in vitro* [45]. Likewise, *L*. *plantarum* grew on gentobiose (Fig 2A, well A6), a rare disaccharide found in the gentian family of plants, however none of these strains utilized raffinose (Fig 2A, well B1), a trisaccharide found in certain vegetables that was shown to support growth of *L*. *plantarum* RYPR1 isolated from traditional Indian fermented beverages [46]. In contrast, only *L*. *rhamnosus* but not *L*. *plantarum* utilized the sugar rhamnose (Fig 2A, well C8).

*Bacillus coagulans*, which was formerly classified as *Lactobacillus sporogenes*, inhabits ecological niches that are more common to lactic acid bacteria than other *Bacillus* spp. [47]. Their pattern of carbon metabolism shows a preference for sugars, similar to the *Lactobacillus* species. Comparing the genomes of probiotic strains of *B*. *coagulans* to *B*. *subtilis* has provided insight into unique or shared properties (i.e. spore formation) [48]. In contrast, *B*. *subtilis* preferentially utilized amino acids rather than sugars. Five amino acids were utilized only by *B*. *subtili*s: L-alanine, L-arginine, L-aspartic acid, L-glutamic acid, and L-histidine (Fig 2B, wells E3-E7), and these wells turned positive (purple) more rapidly than some sugar wells. As a soil microbe subject to nutrient limitation in the environment, *B*. *subtilis* is known to secrete many hydrolytic enzymes and possess greater metabolic versatility [49], which is reflected in its Biolog profile.

We observed strain-level differences in tolerance to environmental stressors such as salt tolerance (Fig 2C, wells B10-B12) among the *Lactobacillus* strains. *B*. *subtilis* was the most salt-tolerant of the species investigated, growing at up to 8% NaCl (Fig 2C, well B12). Strains of both *Bacillus* species could tolerate acidic conditions (pH 5), however none of the *L*. *plantarum* or *L*. *rhamnosus* isolated from probiotics showed growth at pH 5 (Fig 2C, well A12). Only the *L*. *plantarum* strain isolated from a fruit fly gut grew in well A12. In addition to the antibiotics tested on the Biolog plate (Fig 2C, wells D10 through H10), a subset of the probiotic isolates was tested for sensitivity to 12 antibiotics using the disc diffusion (Kirby-Bauer) method (Fig 2D). Antibiotic sensitivity was determined by measuring the zones of growth inhibition around each antibiotic disc and comparing the diameter to a reference table [28]. The antibiotic susceptibility profiles of *L*. *rhamnosus* vs. *L*. *plantarum* were slightly different (Fig 2D). Like most lactobacilli, they are naturally resistant to vancomycin (well F10) due to absence of D-ala in the peptide crossbridge of their cell walls [50], and *L*. *plantarum* strains also displayed resistance to ciprofloxacin. The three *L*. *rhamnosus* strains tested were susceptible to penicillin. *B*. *subtilis* was sensitive to more of the antibiotics than the lactobacilli (Fig 2C and 2D), with strain 8 resistant to only one antibiotic, bacitracin (Fig 2D).

For each bacterial species, we counted the number of Biolog assay wells showing a variable result (disagreement of positive/negative, positive/half, or negative/half) among the probiotic isolates (S5 Table). Strain-specific variation was observed as follows: for *B*. *coagulans*, 21 wells (22%); *B*. *subtilis*, 23 wells (24%); *L*. *plantarum*, 15 wells (16%); and *L*. *rhamnosus*, 32 wells (34%) (S5 Table).

The Biolog system is also capable of identifying yeasts using specific YT plates and the YT database. After optimizing the incubation time and temperature (72 hours at 26#x00B0;C), we identified yeast strains isolated from four probiotic drinks, one probiotic supplement and the surface of a kale leaf with Biolog YT (Table 2, Fig 2E). Three of these (strain codes 21, 22, 25) came from products labelled as containing *Saccharomyces boulardii*, and the correct identification was obtained for two of those three. The metabolic utilization patterns of 35 carbon sources were compared for these probiotic yeasts and are summarized in Fig 2E. The sugars alpha-D-glucose (well C2), turanose (well B12), and maltose (well B3) had clear positive reactions for the all *S*. *boulardii* isolates. Inulin (well A12), a common prebiotic fiber, was utilized by several strains, although the reference pattern for *S*. *boulardii* used in the Biolog database is negative for inulin (Fig 2E). Notably, there was variation in galactose utilization (well C3), a trait proposed previously to distinguish *S*. *boulardii* strains from *S*. *cerevisiae* [51]. Variability in gene clusters for utilization of galactose and palatinose (well B7) was reported in a comparative genomics investigation of probiotic strains of *S*. *boulardii* [52]. The environmental yeast isolate from the kale leaf had pink pigmented colonies, did not belong to the genus *Saccharomyces*, and displayed a unique phenotypic pattern utilizing a wider range of carbon sources (Fig 2E). This contrasting pattern may be reflective of its different ecological niche (leafy green vegetable, versus the original habitat of *S*. *boulardii* on the tropical fruits lychee and mangosteen) [51].

### Analysis of probiotic product labels

One objective of this project was to estimate the percentage of probiotic products currently on the market that list the specific strain ID of the bacterial or yeast species on the ingredient label. Of the products selected for this study, 7/21 (33%) listed the strain ID. To sample the distribution of products with and without strain-level identifications listed on the label, we searched products available from four sources: a major online retailer, two drugstore chains, and a retail superstore. In each, product labelling was evaluated by reading the ingredient label of each unique product and recording whether or not the label listed an alphanumeric strain ID (often the patented name of the strain) after the genus and species. Table 3 summarizes the resulting product counts sampled from four retail sources. Our approximation found that an average of 49% (ranging from 34–69%) of products contained specific strain information on the label.

**Table 3:** Strain identifications listed on probiotic product labels.

Store	# products checked	# products with Strain ID	% with Strain ID
Major online retailer	121	41	34%
Drugstore 1	28	13	46%
Drugstore 2	21	10	48%
Retail superstore	26	18	69%

**Fig 4:**
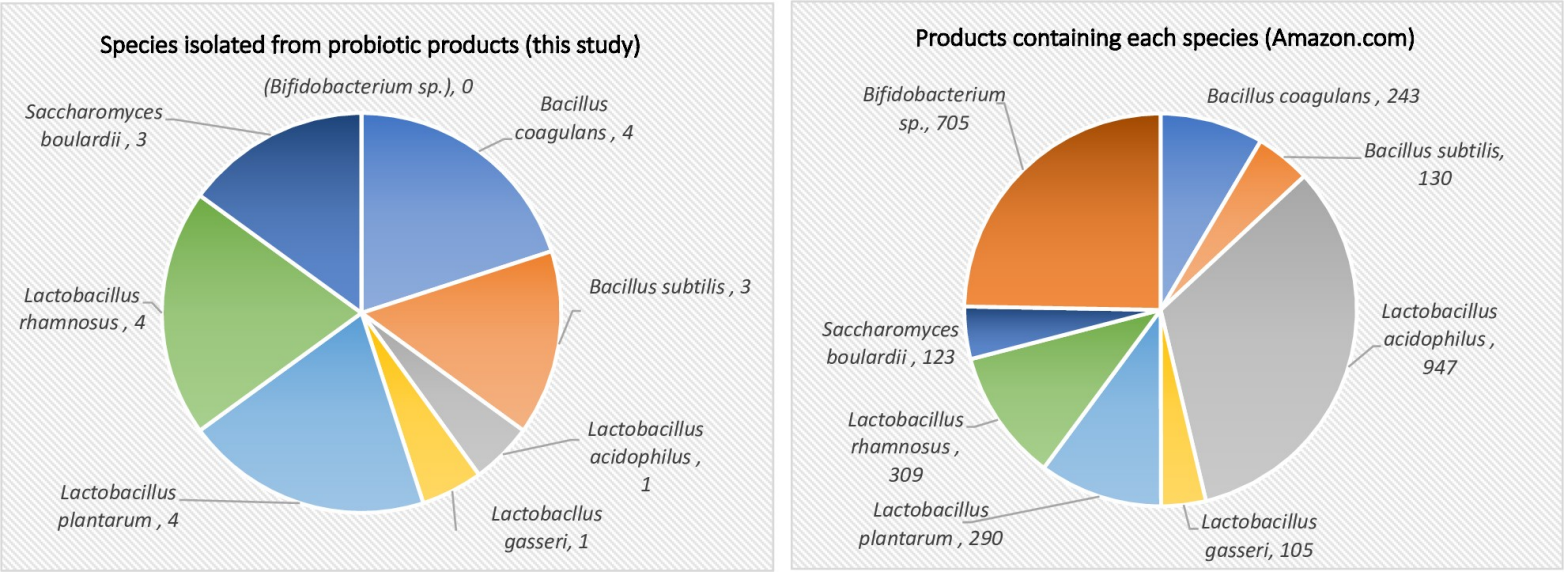
Species in this study and abundance in products for sale by major online retailer. (A) Left: Bacteria and yeast isolated from probiotic products in this study (total = 20). Right: Number of products listed on Amazon.com containing the selected species of probiotic microbes.

Lastly, to provide additional context and approximate the abundance of each species of probiotic sold in products on the market, we searched Amazon.com using the species name and keyword “probiotic.” Fig 4 shows the number of products listed for sale on Amazon.com alongside the breakdown of species investigated in this study.

This study was somewhat representative of the distribution of common probiotic species in commercial products, with exceptions. *Bifidobacterium* species, a major group of probiotic bacteria, were excluded from the current study due to technical challenges of culturing anaerobic bacteria. Similarly, the microaerophilic *L*. *acidophilus* displayed poor growth and this species was underrepresented in this study compared to its wide availability in probiotics. Nevertheless, the species isolated in this work were quite prevalent in this estimation, with both *L*. *plantarum* and *L*. *rhamnosus* listed in roughly 300 products, *B*. *coagulans* in 243 products, and *B*. *subtilis* and *S*. *boulardii* listed in over 120 products each (Fig 4).

## Conclusions

This study highlights that labeling of probiotic products with only the species name may not provide sufficient information about the strain-level diversity in these products. We found that on average, roughly half of the probiotics examined had the specific strain listed on the label, which varied considerably by store (Table 3). Adding strain information to labels would allow consumers and/or healthcare providers to more readily evaluate clinical studies of the probiotic’s effects for specific indications [15]. From survival in the GI tract (by tolerance to acidic pH and bile salts), to adhesion to intestinal cells, to competition with pathogens and production of bioactive compounds, the capacity and efficiency to perform these functions is often strain-dependent [33][53]. While it is well known that strain-level differences occur in the probiotic properties of microorganisms [6][14], our study showed that considerable variability in metabolism and environmental stress tolerance exists between these strains, with between 16–34% of the phenotypic assay wells yielding variable results. Microbial physiology is linked to “probiotic” phenotypes, because the food sources and molecular cues that cells encounter in their environment often directly regulate the expression of proteins and metabolites (or community-level behavior such as aggregation and biofilm formation) that confer the probiotic’s beneficial effect. While this study did not explicitly measure probiotic properties, testing probiotic characteristics would be a logical continuation of this research, and whole-genome sequencing could be used to identify the genetic basis of strain-specific differences.

Many of the carbon sources evaluated with the Biolog assay ultimately contribute to bacterial survival in the GI tract. Examples of the relationship between nutrient sources and bacterial probiotic phenotypes include: increased resistance of *L*. *plantarum* to gastric juices when grown with pectin or inulin compared to glucose [45]; differences in cell surface hydrophobicity, cell surface protein and exopolysaccharide production of *L*. *rhamnosus* grown on fructose, mannose, or rhamnose [54]; and increased the adhesion of *Lactobacillus acidophilus* to mucin or intestinal cells in the presence of fructooligosaccharides (FOS), cellobiose, or polydextrose [55]. The prebiotic cellobiose was shown to change surface layer proteins and increase auto-aggregation in two *Lactobacillus* strains [56]. In another example, plant glucosides from fruit are metabolized by *L*. *acidophilus*, which then secrete aglycones that exert beneficial effects on the host [57]. The wide range of polysaccharides used by *B*. *longum* species has been proposed to aid in their success as early colonizers of the infant gut [58].

In the complex ecosystem of the digestive tract, metabolic profiling of individual microbes and microbial communities can help draw the link between prebiotics, probiotics, the gut microbiome and overall health [59]. Using culture-based microbiology to investigate common probiotics, our work illustrates that metabolic profiling approaches can differentiate between strains from different products. This study highlights strain-specific differences among commercially available probiotic microbes, underlining the importance of accurate labeling to empower consumers to find clinical evidence behind each strain’s beneficial effects.

## Supporting information

S1 Table Probiotic product names and purchase locations. List of products included in the study.
(XLSX)

S2 Table 16S Sequencing results. Top-scoring nucleotide BLAST hit of each bacterial isolate. Near full-length 16S gene sequences were cleaned and trimmed with Geneious software, then “blastn” searched against the NCBI non-redundant nucleotide database (nr). The top-scoring BLAST hits for both the forward and reverse sequences are shown, along with Bit score, E-value, % identity, sequence length, and summary output from the Geneious results file.
(XLSX)

S3 Table Biolog identification. Method details and SIM scores. Detailed experimental conditions for the Biolog identifications are listed, including growth medium, incubation time and temperature, and Biolog inoculating fluid used. Similarity (SIM) scores are based on how closely the pattern of positive wells in the Biolog plate of the isolate matches the reference pattern in the database (GenIII for bacteria, YT for yeast), and an identification is provided by the software if the SIM score is >0.5.
(XLSX)

S4 Table MALDI-TOF mass spectrometric identification. Method details and confidence scores. The formic acid protocol was used first to prepare the bacterial colonies for MALDI-TOF. For bacterial isolates that were not successfully identified with the formic acid method, the protein extraction or modified protein extraction method was used. Each mass spectrum was assigned a confidence interval score based on comparison to SuperSpectra in the SARAMIS database. Taxonomic names are assigned by the software if confidence levels are >75%.
(XLSX)

S5 Table Biolog assay wells with variable results within species. The result from each reaction well of the GenIII Biolog plate was compared across the strains of each species isolated from probiotic products. Disagreement is defined as one or more strains of that species showing a different reaction result from others: Positive (P), negative (-), or borderline ('h').
(XLSX)
